# Protocol for the trial to establish a causal linkage between mycotoxin exposure and child stunting: a cluster randomized trial

**DOI:** 10.1186/s12889-020-08694-6

**Published:** 2020-05-01

**Authors:** Erica Phillips, Francis Ngure, Laura E. Smith, Edna Makule, Paul C. Turner, Rebeca Nelson, Martin Kimanya, Rebecca Stoltzfus, Neema Kassim

**Affiliations:** 1 Independent Research Consultant, Arusha, Tanzania; 2 Department of Epidemiology and Environmental Health, School of Public Health and Health Professions, University at Buffalo, Buffalo, NY USA; 3 Department of Food Biotechnology and Nutritional Sciences, School of Life Science and Bio-Engineering, The Nelson Mandela African Institution of Science and Technology (NM-AIST), P.O.Box 447, Arusha, Tanzania; 4 MIAEH, School of Public Health, University of Maryland, College Park, MD 20740 USA; 5 School of Integrative Plant Science, Plant Pathology and Plant-Microbe Biology Section, Cornell University, Ithaca, USA; 6 Goshen College, 1700 S. Main Street, Goshen, Indiana 46526 USA

**Keywords:** Stunting, Aflatoxin, Mycotoxin, Nutrition

## Abstract

**Background:**

The number of stunted children has fallen globally but continues to increase in Africa. Stunting is estimated to contribute to 14–17% of child deaths under 5 years of age and is a risk factor for poor cognitive and motor development and educational outcomes. Inadequate dietary intake and disease are thought to be the immediate causes of undernutrition and stunting. However, improving infant diets through complementary feeding interventions has been shown to only modestly reduce stunting. Multiple observational studies demonstrate a dose response relationship between fetal and post-natal aflatoxin exposure and reduced linear growth.

**Methods:**

This community-based cluster randomized trial will measure the effect of a reduced aflatoxin diet on length-for-age Z scores at 18 months in central Tanzania. All 52 health facilities in the Kongwa District of Dodoma Region were randomized into two groups. Starting at 6 months of age, participants in the intervention group receive a low-aflatoxin pre-blended porridge flour containing maize and groundnut (ratio 4:1 respectively) and low-aflatoxin groundnut flour, whereas in the control group the same porridge mix and groundnut flour are promoted through education but acquired by the household. Both groups will receive the same infant and young child feeding education and a thermos flask. A total of 3120 infants between 6 weeks and 3 months of age will be recruited into the study over 1 year. Data will be collected four times – at recruitment and when the infants are 6, 12 and 18 months of age. In a cohort of 600 infants, additional data will be collected at 9 and 15 months of age. The primary outcome is length-for-age at 18 months. Secondary outcomes include the Z scores for weight-for-age, middle upper arm circumference and head circumference, and the blood biomarker aflatoxin-albumin in the full sample, with the urine biomarker aflatoxin M1 analyzed in the cohort only.

**Discussion:**

Better understanding the etiology of childhood stunting can lead to more appropriate interventions and policies to further reduce linear growth faltering and meet the Sustainable Development Goals.

**Trial registration:**

NCT03940547, (April 24, 2019).

## Background

### Background and rationale

In 2018, 21.9% of children under 5 were stunted globally, the heaviest burden in South Asia and Eastern Africa [1]. Stunting is estimated to contribute to 14–17% of child deaths under 5 years of age and is associated with poor cognitive and motor development and educational outcomes [2–6]. The etiology of stunting has long been attributed to inadequate dietary intake and gastrointestinal illness, including diarrhea [7]. However, multiple reviews and recent large trials of nutrition education, complementary feeding programs and water, sanitation and hygiene (WASH) interventions to reduce stunting have shown modest or no effects on linear growth in food insecure populations, those who would theoretically benefit the most from such intervention [8–13]. In three systematic reviews, the effect of provision of complementary foods with or without education on LAZ was small to moderate, with effect sizes ranging from 0.10–0.39 [9, 10, 14]. It is therefore plausible that other factors contribute to stunting, including mycotoxin exposure [15–17].

Mycotoxins, including aflatoxin (AF) and fumonisin (FUM) are naturally occurring secondary metabolites produced by fungi that colonize foodstuffs. Approximately 0.5 billion people, mainly living in developing countries, are at risk of being chronically exposed to dietary AF at high levels [18]. Maize and groundnut, two key staples in eastern and southern Africa, are particularly vulnerable to contamination by AF; maize is vulnerable to contamination by FUM as well. Exposure to AF in low-income populations, especially those in rural sub-Saharan Africa, is particularly problematic because the diet is monotonous, largely based on AF-prone crops, and grown and stored under conditions that predispose them to contamination.

AF are proven human liver carcinogens and intermittent outbreaks of extremely high levels of AF consumption cause acute and often fatal liver toxicosis [19, 20]. The effects of less extreme AF exposure during critical periods of early-life development are not understood, although AF and FUM are suspected of contributing to stunting. The pathways between AF consumption and growth impairment are not known, however one plausible mechanism is propagation of environmental enteric dysfunction (EED), a sub-clinical disorder that damages the lining of the small intestine, causing mucosal damage and impairing its function [15, 17, 21]. EED can lead to stunting by causing nutrient malabsorption, growth hormone resistance, and impaired bone growth and remodeling [22]. It is hypothesized that AF and FUM share a pathway that overlaps with EED, which may include interactions with both gastrointestinal infection and liver toxicity leading to impaired protein synthesis or homeostatic function [16]. Additional mechanisms for AF driven growth faltering have been suggested but remain unproven [23, 24].

Multiple observational studies have shown an association between fetal and post-natal aflatoxin exposure and linear growth [25–27]. In rural Benin and Togo, the concentration of the blood biomarker, serum aflatoxin albumin (AF-alb), was associated with growth deficiencies among young children from 9 months to 5 years, as demonstrated by significant dose-response relationship with both length-for-age (LAZ) and weight-for-age Z scores (WAZ) [25]. In a subsequent longitudinal study in rural Benin, infants in the highest quartile of AF- alb, compared to the lowest quartile were 1.7 cm shorter after 8 months [26]. A longitudinal survey of AF exposure during pregnancy and via early introduction of complementary foods examined the relationship between exposure and infant growth from birth to 52 weeks. A significant inverse relationship between maternal AF exposure and infant growth velocity was observed [27]. A more statistically significant effect on growth was seen when models included early infant exposure (measures at week 16 years of age) in addition to maternal AF, suggesting multiple exposure windows may be possible to affect and or mitigate AF induced growth effects.

An observational study in Tanzania assessed the effects of AF and FUM exposure on attained LAZ at 12 months [28]. Urinary FUM was negatively associated with LAZ (*p* = 0.01), with children in the highest quartile of exposure 1.8 cm shorter than children in the lowest quartile of exposure; the negative association between AF-alb and LAZ did not reach statistical significance, however AF-alb levels were lower compared to studies in West Africa described above. The MAL-ED study in Nepal also did not find an association between AF consumption and stunting, but it too was conducted in an area of lower AF exposure and lower prevalence of stunting compared to West Africa [29].

Most of these observational studies have been small with varying assessment and analytic methods used between them, making statistical comparisons between them difficult. Yet taken together, these results suggest a potential threshold of exposure above which AF driven growth faulting occurs.

One randomized trial has assessed the effect of reduced AF consumption on linear growth [30]. The study did not find an improvement on LAZ at 18 months in the intervention group, despite a 27% decrease in the blood biomarker, AF-alb [31]. However, there was a significant difference in LAZ at midpoint, even though AF-alb did not differ between groups at midpoint. Overall AF-alb was modest in this study when compared to data from West Africa [20–22]. Confounding interpretation of these results, 27 % of recruited infants were not included in the analysis of the primary outcome and 65% of AF-alb samples were analyzed due to loss of follow-up.

To fill current gaps in knowledge about the effect of AF exposure on child growth we designed a community-based cluster randomized trial to assess the effect of a low AF diet on linear growth. Our intervention was designed to target feeding behaviors for the index child and to specifically isolate the effect of AF consumption between the intervention and control groups. We will collect contextual data about levels of AF contamination in infant foods and multiple human biomarkers and assess feeding practices and adherence to our intervention at multiple time points to better understand the pathways between AF ingestion and growth retardation.

## Methods

### Aims

This two-group cluster randomized trial (CRT) is designed to test the hypothesis that provision of a complementary food porridge flour and separate groundnut flour, both with very low levels of AF (AFB1 < 5 ppb) will be associated with better linear growth in infants between 6 and 18 months of age compared to infants who consume complementary porridge and foods made with similar ingredients and groundnut flour, but acquired by the household.

The primary objective of the trial is to measure the effect of a low-AF diet on length-for-age Z scores at 18 months. Select secondary outcomes are to:

1. Determine the effect of an intervention to reduce AF exposure on LAZ scores at 12 months and stunting at 12 and 18 months
2. Determine the effect of an intervention to reduce AF exposure on WAZ and underweight at 12 and 18 months
3. Examine the effect of an intervention to reduce AF exposure on mid-upper arm circumference (MUAC) and head circumference at 12 and 18 months
4. Assess the effect of an intervention to reduce AF exposure on the blood biomarker AF-alb at 12 and 18 months
5. Assess the effect of an intervention to reduce AF exposure on the urinary biomarker AFM1 in a cohort of 600 infants at 9, 12, 15 and 18 months

A full list of all 14 secondary outcomes is included in Appendix 1.

### Study setting

The trial is being conducted in Kongwa District, Dodoma Region of Tanzania. This district was selected because stunting in the region is 37%, slightly above the national average, and there were 8000 live births in 2017, enough to support the trial’s sample size [32]. The unit of randomization is a health facility. All 52 health facilities in the district (1 district hospital, 4 health centers and 46 dispensaries) were randomized into the control or intervention group. Two community health workers (CHWs) per health facility were selected by the management of the facility to be employed part-time by the research project. These CHWs were trained by Tanzanian Food and Nutrition Center trainers in March of 2019 in infant and young child feeding (IYCF) and lead IYCF education sessions for trial mothers in their communities. The majority of data collection, including collection of all biological samples, is performed at the health centers.

### Recruitment

Recruitment into the trial is conducted through each health facility using Expanded Program on Immunization (EPI) attendance records. EPI coverage is > 95% in Kongwa District [33]. All 52 facilities are visited in a “round” of recruitment over 5–6 weeks, with recruitment ongoing for one calendar year. The names of the infants who attended the 42 day EPI visit since the previous round’s visit are recorded from the facility’s register. In sequential order by visit date, mothers of these infants are visited in their homes, screened for eligibility and invited into the study, until the target number of women agree to participate. The informed consent procedure is performed by a trained data collector at the time of recruitment.

### Inclusion and exclusion criteria

Inclusion criteria:

1. Babies > 6 weeks old and < 4 months old, who seek EPI from a randomized health facility and reside in Kongwa District

Exclusion criteria, assessed at recruitment and again at the 6-month visit:

1. A baby with a disability that preclude normal feeding and swallowing
2. A parent who refuses to consent to assigned intervention
3. An infant who has shown signs of a potential groundnut allergy (assessed the first time mother reports groundnut consumption)
4. An infant who is a twin
5. The mother plans to travel for more than two months at or after the randomized intervention begins
6. A mother below 16 years of age

### Formative research

The trial intervention was designed following multiple years of formative research in Kongwa District. We collected food samples across the district to overlay AF contamination of foods with stunting and birthrates using GIS. In the year prior to the trial launch, we collected 387 food samples at two time points from 115 households in 24 villages across Kongwa, which revealed frequent AF contamination of both maize and groundnuts (Ngure et al., manuscript in prep). The levels of AF contamination for groundnut ranged between 0.6–3600 ppb, with 56% of samples > 20 ppb. For maize, 6% of samples were > 20 ppb with a range between 0.4–55.5 ppb. Other foods reported consumed by infants, included millet, finger millet, rice, sorghum and sunflower seeds, of which 5% had AF > 20 ppb. The AF contamination in these foods was similar in frequency and levels to previous studies linking AF to growth faltering in West Africa and Zimbabwe [27, 34, 35].

Five focus groups and recipe trials were conducted to test the acceptability of the proposed pre-blended porridge flour of 3 or 4 parts maize to 1 part groundnut, the advice of the District Nutrition Officer, and to inform IYCF educational messages. From these discussions, two short-term trials of improved practices (TIPS) were conducted as proof of concept of the main trial intervention [36]. The first TIPS tested mothers’ acceptability and responsiveness to the control condition, promotion of a pre-blended porridge flour made of maize and groundnut and the addition of groundnut to relishes or sauces consumed by the infant (manuscript in prep). Briefly, in this first trial of 17 days, 66% of infants at baseline and 71% of infants at follow-up (*n* = 35) consumed groundnut on the previous day. Slightly more mothers reported adding groundnut in porridges and vegetable relish at follow-up than baseline, indicating a willingness to incorporate educational messages into infant feeding practices. The second trial tested mothers’ acceptability of the intervention condition (provision of a pre-blended porridge flour). Three day consecutive urine samples were collected both at baseline and follow-up in all infants (*n* = 37). Provision of low-AF pre-blended porridge flours reduced the prevalence of detectable urinary AFM1 by 81% in following provision of AF-free lishe for 7–10 days, indicating high acceptability.

Based on these findings, we refined our trial intervention to promote and provide pre-blended flours at a ratio of 4 parts maize to 1 part groundnut, the median ratio of practices found by mothers in the 24 h recalls collected in the mini-trials. This decision will reduce the potential for a difference in dietary intake between groups and reduce ethical concerns of increasing groundnut consumption in the control group. Secondly, the trial will use a separate low AF groundnut flour in the intervention group, in addition to pre-blended maize and groundnut flour, as groundnut was commonly used to flavor foods consumed by the infant and the household and is a potential source of AF exposure. Finally, a thermos flask will be provided to all trial mothers to hygienically store cooked porridge throughout the day.

### Trial intervention

Beginning when the index infant is 6 months of age, data collectors and research staff meet with mothers at each health facility one time per month to collect data and deliver the research supplies to participants. Following data collection, mothers in the intervention group receive pre-blended porridge flour in sealed plastic containers. Fifty grams/day is provided for 6–8 month olds, 60/day grams for 9–11 months and 75 g/day for 12–18 month olds, and containers contain an additional buffer of 10–15 g per day to account for any loss or sharing in the household. These amounts were calculated to provide all of the caloric needs for 6–8 month olds and progressively less calories and macronutrients for older infants as their diets are more diverse, while taking into account local feeding practices. The intervention group will also receive 1 kg of low-AF groundnut flour each month for between 6 and 18 months. Mothers in the control group receive skin lotion every month. At the 6 month visit all mothers are given a thermos flask and a plastic scoop to measure the appropriate amount of porridge flour (Table 1).

**Table 1:** Intervention activities

**Intervention Group**	**Control Group**
Infant feeding education: Breastfeeding Dietary diversity Feeding frequency Hand washing	Infant feeding education: Breastfeeding Dietary diversity Feeding frequency Hand washing
Behavior change communication on use on use of porridge flours: Timing of introduction, frequency of feeding, density and composition Promotion of 4:1 ratio of maize meal to groundnut powder Promotion of use of groundnut flour in infant foods	Behavior change communication on use of porridge flours: Timing of introduction, frequency of feeding, density and composition Promotion of 4:1 ratio of maize meal to groundnut powder Promotion of use of groundnut flour in infant foods
Provision of pre-blended porridge flours and groundnut flour monthly Provision of thermos flask and scoop at 6 month visit	Provision of skin lotion monthly Provision of thermos flask and scoop at 6 month visit

Between the time of recruitment (between 6 weeks and 3 months of age) and the start of the randomized intervention at 6 months of age, all trial mothers are invited to a 4 and 5 month IYCF education session led by local CHWs, with a focus on promotion of exclusive breastfeeding, hand washing, and preparing to feed at 6 months, based on Government of Tanzania education materials [37]. Between 8 and 11 months of age, all mothers are invited to two additional infant and young child feeding education sessions that focus on age-appropriate feeding frequency, diet quality and diversity, and the importance of continued breastfeeding. At the monthly pick-up of the flours or skin lotion, the CHWs lead a short education session to reinforce the main IYCF messages taught at the longer sessions with additional messages about hygienic storage of all infant food flours. The only difference in messages between groups is that intervention group mothers are advised that the provided porridge flour is for the index child only.

There is no blinding in this study, as all parties will be able to see what is received by the mother – pre-blended flour and groundnut flour or lotion.

### Complementary Food production

Critical to the trial’s success is the production of low-AF porridge and groundnut flours. We have partnered with a Tanzanian flour miller to produce the research foods. Procurement of maize and groundnut will be performed collaboratively between the researchers and the miller. *Pendo* groundnut variety, the type commonly consumed in Kongwa, will be sourced mainly from Dodoma region to mimic local consumption and quality. A multiple stage sampling and testing quality control procedure of pre- and post-processed flours was designed by the research staff. Upon receipt, all groundnuts are sorted to reduce AF. Prior to processing, multiple samples will be drawn from maize and groundnut lots and tested for moisture content and AF. The final products will be accepted if AF is ≤5 ppb and FUM is ≤2 ppm [38]. All processed flours will be packaged for monthly consumption to ensure no risk of spoilage or rancidity.

### Adherence to the intervention

Accurately measuring adherence of a complementary and supplementary food distribution program is difficult [39–41]. Self-report, including calculation of a disappearance rate tends to overestimate adherence, but does not demand high resources [39]. Direct observation and analysis of a biomarker, if available, can be more reliable but also time consuming and expensive. In the absence of a “perfect” method of assessing adherence, this trial will use multiple methods: analysis of the urinary biomarker AFM1, calculation of a disappearance rate based on maternal report and maternal interviews about infant feeding practices.

Urinary AFM1, a metabolite of aflatoxin, is a validated biomarker that represents recent exposure in the past 2–3 days [42]. AFM1 will be used to measure short-term adherence in a selected cohort of infants (described in “Study timeline” below) at 9, 12, 15 and 18 months. Adherence to the recommended frequency of cooking and amount of pre-blended porridge flour and groundnut flours cooked will be measured by calculating the daily disappearance rate (# of kilograms provided - # kg remaining/number of days since previous distribution) of the provided foods in the cohort at 12 months. Further probing to assess feeding practices will include reported consumption by the index child in the previous 24 h and previous week, acceptance of the products by the child, sharing by other family members and neighbors, how leftovers are handled, the time it takes to complete a package of foods and maternal acceptance of cooking and the flavor of the foods. In both groups at all time points, mothers will be asked about porridge feeding practices (not exclusive to the provided porridge flour), including the ingredients used in the porridge, consistency of cooked porridge and frequency of feeding.

### Implementation Fidelity

Implementation fidelity is “the degree to which programs are implemented as intended” [43]. Monitoring the fidelity of a trial intervention provides contextual interpretation of the trial results and is useful for both internal and external validity [44]. For example, if the trial finds no evidence of effect, was the biological hypothesis flawed, the intervention poorly targeted to the biological pathway (theory failure) or was this due to a poorly implemented intervention (program failure)? Alternatively, if the trial finds evidence of an effect on child growth can it be demonstrated that the intervention was implemented as designed and is likely to be successful in an alternate setting? A Program Impact Pathway (PIP) approach will be used to monitor the intervention delivery and maternal uptake [45, 46].

Prior to the start of the trial, we reviewed IYCF best-practices and IYCF theory-based program evaluations to design a schematic PIP highlighting critical actions and behaviors that could drive or moderate intervention success [45, 47, 48]. We then used this PIP to identify key indicators to be tracked throughout the trail. These include: 1) timely delivery, quantity and quality of the project foods, skin lotion, thermos flask and scoops, 2) timely delivery of and maternal attendance of CHW-led education sessions, and 3) maternal uptake of promoted IYCF behaviors.

There are three to four full time staff, including “Implementation Officers” and a logistician, who are responsible for delivery of the trial products. Receipt of trial products will be recorded electronically. CHWs keep paper records of IYCF sessions held and attendance at these sessions, which will be reviewed and submitted to the Implementation Officers on a monthly basis. Uptake of behaviors is from self-report by the mother at all survey time points.

### Study timeline

Mothers will meet with data collectors four times throughout the study (Table 2). The first survey will be conducted at recruitment, when the index infant is between 1.5–3 months of age. The second survey will take place when the infant is 6 months of age, the third at 12 months and the fourth and final survey at 18 months of age. We will also include a cohort of 600 infants (300 per group) who will be visited at 9 and 15 months, in addition to the standard visits.

**Table 2:** Summary of data collection – location, method, timing and topic

**Research Visit**	**Recruitment**	**6 month**	**9 month**^**a**^	**12 month**	**12 month**^**b**^	**15 month**^**a**^	**18 month**
**Location**	Home	Health Facility	Health Facility	Health Facility	Home	Health Facility	Health Facility
		Randomized intervention begins					Randomized intervention ends
**Data Collected**							
Household composition, socioeconomic status	I						I
WASH practices	I/O				I/O		I/O
Exposure to CHW education; infant morbidity	I	I	I	I		I	I
Access to health care	HC	HC	HC	HC	HC	HC	HC
Household food security and maternal dietary diversity		I		I			I
UNICEF/WHO feeding indicators; Porridge- specific feeding practice		I	I	I		I	I
Adherence to intervention (intervention group only)			I	I	I/O	I	I
24 h recall					I		
Food collection					I		
Anthropometry (length, weight, MUAC, head circumference)		M		M			M
Blood		T		T			T
Urine		T	T	T		T	T

*HC* Health Card, *I* Interview, *M* Measurement, *O* Observation, *T* Test, ^a^Cohort only, ^b^Cohort sub-set

The surveys will assess demographic and economics of the household, infant feeding practices, access to health-care, household food security, maternal dietary diversity, community health worker contact, and water, sanitation and hygiene of the household. At the 6, 12, and 18-month survey points anthropometric assessment of weight, height, MUAC and head circumference will be conducted in all infants. Blood collection will occur at three time points (6, 12 and 18 months) in all infants, and urine collection only from the cohort of 600 infants at five time points (6, 9, 12, 15 and 18 months). In a sub-set of 250 household in the cohort, maize and groundnut samples will be collected, as well as an assessment of adherence to the intervention and 24-h recalls.

### Data collection and management

Twenty-four data collectors will be hired at the peak of the trial. All data collectors will be trained in research ethics and Good Clinical Practices (GCP). Anthropometrists will be trained to measure recumbent length, weight, MUAC and head circumference. Length will be measured with a ShorrBoard© and weight using an ADE M321600 Electronic Floor Scale with mother/baby tare feature, recorded to the tenth decimal.

Survey data will be collected electronically, using hand-held devices (Samsung Galaxy 7, KoBoToolbox Platform), with paper copies provided as back up in case of electronic failure. Data quality checks are built into the electronic version of the survey, for example limiting ranges of responses/measurements to those that are within a credible range and consistency checks between questions. The Data Quality Supervisor will check all surveys and uploads encrypted data on a daily basis to KoboToolbox. The Research Coordinator in Arusha will monitor enrollment numbers, rejection/non-qualifier numbers, drop-out rates, completed visit rates, anthropometry and biological sample collection success rates each week. The full data set will be encrypted, password-protected and stored on Cornell IRB-approved locations.

### Collection and Management of Biological Samples

Collection of blood and urine will be performed by certified Tanzanian nurses in health facilities following the Standard Operating Procedures. These nurses will be identified and hired in collaboration with the Kongwa District Medical Officer.

Urine samples will be collected with a urine bag, transferred to a sterile container for transport and placed into a cooler box with ice packs. Blood samples will be collected using a vacutainer system with EDTA tubes, placed in a Styrofoam rack then put into a cooler box with ice packs. All samples will contain a barcode for tracking. Samples will be transported to the research lab at the Kongwa District Hospital within 4 hours of collection. The laboratory scientist in Kongwa will record and catalog samples using a hand-held device (Samsung Galaxy 7, KoBoToolbox Platform), then process blood and urine samples upon arrival from the field. Plasma will be isolated and both plasma and urine aliquots will be stored at -40F. Biohazard material and sharps will be discarded through partnership with the Kongwa District Hospital.

### Randomization procedure

To capture the potential range of exposure across the district, all facilities were included in the sampling frame, and their unique catchment area is considered a cluster. The randomization procedure was developed to ensure close balance between the two groups on altitude and number of EPI visits.

Altitude is associated with mycotoxin risk, with 1000–1200 m considered the threshold for drier and colder conditions in which toxin-producing fungi are less likely to thrive. Number of EPI visits was used as a proxy for health facility size, population density and access to resources such as roads and markets.

The following steps were taken to randomized health facilities:

1. Sort the health facilities by low versus high altitude using the median of the district, 1200 m, as the cut-off.
2. Sort the health facilities by size within each altitude category, using 42 day immunization visit attendance data from the most recent year of data (August 2017–July 2018).
3. Group health facilities into successive pairs from the top to the bottom of each altitude category. Half of the pairs within a list were randomly chosen. For the chosen pairs, the first health facility in each pair was assigned to the experimental group and the other health facility assigned to the control group. For the pairs not chosen, the assignments were reversed: the first health facility in each pair was assigned to the control group and the other health facility assigned to the experimental group.

### Sample size calculation

The sample size calculation was estimated to detect a difference of 0.2 LAZ score between the intervention and control groups using the STATA (version 15.1) command for two independent sample means in a CRT. This difference in LAZ is believed to be clinically significant and would therefore motivate public health decision-making.

Using a one-sided test of independent sample means, with a standard deviation of 1.2 Z, type I error of 0.05, and power of 0.90, design effect of 2.0 (justified below) and randomizing all 52 health facilities (26 per group), the total sample size was calculated to be 2322 (1161 infants per cluster). This also assumes a coefficient of variation of .14 for varying cluster size, based on previous year’s data for EPI attendance at 42 days. A conservative estimate of 20% loss to follow-up and infant mortality indicates a total of 2787 infants, or 54 infants recruited per health cluster annually or 4.5 infants per cluster per month is needed. Therefore, if five infants per cluster per month are recruited, that will results in a total of 3120 infants in the study, recognizing that in approximately six of the health facilities, it may be difficult to recruit sufficient numbers based on the size of the population served by the facility.

To estimate the design effect, we identified other cluster-randomized trials with educational and/or food provision interventions delivered through CHWs with LAZ as an outcome [49–51]. Based on these studies, we used an intraclass correlation coefficient of 0.02 to reach our design effect of 2.0. This is in line with the estimated design effects for stunting using population-based surveys in three African countries calculated by Katz [52].

### Cohort selection

Twenty-four health facilities (12 heath facility pairs) were purposively selected to be the cohort sampling frame, based on geographic accessibility of the facilities themselves and their catchment households. At the 6 month visit, when the randomized intervention begins, 2–3 mothers at these facilities are randomly selected into the cohort on a monthly basis by pulling different colored marbles from a bag. If a mother accepts participation in the cohort, there is an additional informed consent procedure.

### Statistical analysis and data analysis plan

All outcomes will be presented using descriptive statistics; normally distributed data by the mean and standard deviation (SD) and skewed distributions by the median and interquartile range (IQR). AF biomarkers and food contamination, usually skewed, will additionally be presented as geometric means and 95% CIs, medians and IQRs. Binary and categorical variables will be presented using counts and percentages. These outcomes will additionally be presented by study group and season.

The primary analysis will compare LAZ at 18 months between the control and intervention groups and will be analyzed using a mixed-model, such as Stata GEE regression procedure, to account for with-in cluster variance. The primary analyses will be intention-to-treat.

Secondary analyses will include:

- Intention-to-treat analyses of human mycotoxin biomarker outcomes
- Analyses of mediation of mycotoxin biomarkers on the primary outcomes, using path analysis and/or regression approaches
- Adjusted estimates of the randomized treatment effects on primary and secondary outcomes, using multiple regression equations to adjust for imbalances between randomized groups, and potentially increase precision of the effect estimate. For this analysis we will include covariates that differ substantially between randomized groups and/or that modify the intervention effect size by at least 10% when included in the model.
- Per-protocol analysis of those with the highest contact with CHWs responsible for delivering the intervention and those who report high adherence to the intervention. In these analyses, we will consider the CHW contact and adherence to the intervention as ordinal variables defined by tertile or quartile of the continuous variables.
- By sub-groups: socio-economic status, quartile, sex, elevation of residence, quartiles of LAZ at 6 months

Trial results will be reported in accordance with the extended CONSORT guidance for cluster randomized trials.

### Missing data

In the main analyses we will include all relevant continuous covariates with less than 10% missing. For categorical variables, we will create a dummy variable for missing. For variables that have substantial missing we will conduct sensitivity analyses with multiple imputation to compare to the main analyses.

### Ethics

This trial has been approved by the Cornell IRB and the Tanzanian National Institute for Medical Research (NIMR). Any protocol amendments have been approved prior to implementation and updated in ClinicalTrials.gov. Written informed consent is asked and obtained by all participants in Kiswahili prior to the start of data collection. Our informed consent forms have been written for a population with limited research experience and research knowledge and contains multiple questions to check for comprehension of the study and the participant’s rights. These forms are written specifically for each group, since randomization is at the cluster (health facility) level, prior to the start of recruitment. Further consent is requested to store biological samples for future use.

All staff, including community health workers who have the most contact with mothers, were trained on the trial’s Unexpected Events Reporting plan to ensure appropriate and timely reporting to the Cornell IRB and NIMR. Survey data will be reviewed for attrition, child morbidity, any reports of protocol deviations on a monthly basis. Participant complaints and expected or unexpected events are received on a case-by-case basis and are reported to the IRB according to our reporting plan.

An independent Data Safety and Monitoring Board (DSMB) was established, consisting of a biostatistician, a pediatrician and a nutritionist. The DSMB will convene three times – once around the time of the study’s launch (completed August, 2019), near the end of recruitment (completed February, 2020) and at the end of the trial. The DSMB will monitor trial data at 6-monthly intervals, beginning in August 2020. The following data are included in the monitoring report, active complaints from mothers about an index infant’s health, care-seeking from clinic, doctor or hospital for baby illness, stunting prevalence (LAZ < − 2), wasting prevalence (WHZ < − 2) and possible severe acute malnutrition (SAM). Infant deaths will also be reported to the DSMB as soon as possible. The DSMB decided not set any stopping rules.

### Dissemination policy

The Bill and Melinda Gates Foundation, the funder of this trial, requires an open access data policy. Therefore, all manuscripts from this funded work will be open access with the data underlying the published research results. More on this policy can be found: https://www.gatesfoundation.org/how-we-work/general-information/open-access-policy

Additionally, we will present our results with the Kongwa District Council, as well as a final report to NIMR and the Tanzanian Commission for Science and Technology (COSTECH). We are not authorized to share any results with the participants or communities directly but can request the local government to share only summary results (not results by health facility) to assure confidentiality is upheld.

## Discussion

Prior to initiation of field work, the researchers performed a literature review about the ethics of conducting a trial focused on mycotoxin exposure, studying general bioethics and relevant randomized trials in environmental health and HIV treatment in low and middle income countries (Phillips et al., manuscript in prep). The design of this study attempts to balance risk and benefit to all participants.

Alongside the trial, this project is also working towards means to reduce AF and FUM in foods by innovating various sorting technologies appropriate for low-input food chains. We are performing extensive size and visual sorting experiments of maize and groundnuts to reduce of contamination and lead to improved grain sorting techniques to reduce AF and FUM contamination.

The results of this trial will strengthen the understanding the effect of AF exposure on growth in infants and young child. This study has the potential to provide policy makers with valuable information about the potential contribution of AF on growth retardation and to ultimately benefit the most vulnerable populations where growth deficiencies persist.

## Data Availability

Not applicable.

## Appendix

### Appendix 1: List of all secondary outcomes

1. To determine the effect of an intervention to reduce AF on LAZ scores at 12 months and stunting at 12 and 18 months
2. To determine the effect of an intervention to reduce AF on weight for age Z scores (WAZ) at 12 months and 18 months and underweight at 12 and 18 months
3. To examine the effect of an intervention to reduce AF on mid-upper arm circumference and head circumference at 12 and 18 months
4. To assess the effect of an intervention to reduce AF on the blood biomarker AF-alb at 12 and 18 months
5. To assess the effect of an intervention to reduce AF on the urinary biomarker AFM1 in a cohort of 600 infants at 9, 12, 15 and 18 months
6. To assess the effect of an intervention to reduce AF on the urinary biomarker FUM in a cohort of 600 infants at 9, 12, 15 and 18 months
7. To describe the relationship between recent consumption AF in food and AFM1 in urine in a cohort of infants at 12 months
8. To determine the relationship between FUM in food and urinary FUM biomarkers in a cohort of infants at 12 months
9. To determine how demographics, geography and climate modify spatial measures of food consumption and food contamination with AF and FUM
10. To determine how pre- and post-harvest practices influence contamination of food with AF and FUM
11. To use blood and urinary biomarkers to hypothesize mechanisms of AF-induced stunting at 12 and 18 months, including a threshold of exposure
12. To identify early life food and cultural behaviors that may influence food choices and AF exposure
13. To investigate role AF and FUM co-exposure on stunting, LAZ, underweight and WAZ at 12 and 18 months
14. To determine the effect of an intervention to reduce AF on inflammatory markers (CRP and TNFa) at 18 months of age

## Abbreviations

AF: Aflatoxin
AF-alb: Aflatoxin-albumin
AFM1: Aflatoxin M1
CHW: Community health worker
COSTECH: Tanzanian Commission for Science and Technology
CRT: Cluster randomized trial
DSMB: Data Safety and Monitoring Board
EED: Environmental enteric dysfunction
EPI: Expanded Program on Immunization
FUM: Fumonisin
GCP: Good Clinical Practices
IRB: Institutional Review Board
IQR: Interquartile range
IYCF: Infant and young child feeding
LAZ: Length-for-age
MUAC: Middle-upper arm circumference
NM-AIST: The Nelson Mandela African Institution of Science and Technology
NIMR: National Institute for Medical Research
PIP: Program Impact Pathway
SD: standard deviation
WASH: Water, sanitation and hygiene
WAZ: weight-for-age Z scores
